# Charles Bonnet Syndrome: Evidence for a Generative Model in the Cortex?

**DOI:** 10.1371/journal.pcbi.1003134

**Published:** 2013-07-18

**Authors:** David P. Reichert, Peggy Seriès, Amos J. Storkey

**Affiliations:** 1 Institute for Adaptive and Neural Computation, University of Edinburgh, Edinburgh, United Kingdom; 2 Department of Cognitive, Linguistic & Psychological Sciences, Brown University, Providence, Rhode Island, United States of America; École Normale Supérieure, College de France, CNRS, France

## Abstract

Several theories propose that the cortex implements an internal model to explain, predict, and learn about sensory data, but the nature of this model is unclear. One condition that could be highly informative here is Charles Bonnet syndrome (CBS), where loss of vision leads to complex, vivid visual hallucinations of objects, people, and whole scenes. CBS could be taken as indication that there is a *generative model* in the brain, specifically one that can synthesise rich, consistent visual representations even in the absence of actual visual input. The processes that lead to CBS are poorly understood. Here, we argue that a model recently introduced in machine learning, the deep Boltzmann machine (DBM), could capture the relevant aspects of (hypothetical) generative processing in the cortex. The DBM carries both the semantics of a probabilistic generative model and of a neural network. The latter allows us to model a concrete neural mechanism that could underlie CBS, namely, homeostatic regulation of neuronal activity. We show that homeostatic plasticity could serve to make the learnt internal model robust against e.g. degradation of sensory input, but overcompensate in the case of CBS, leading to hallucinations. We demonstrate how a wide range of features of CBS can be explained in the model and suggest a potential role for the neuromodulator acetylcholine. This work constitutes the first concrete computational model of CBS and the first application of the DBM as a model in computational neuroscience. Our results lend further credence to the hypothesis of a generative model in the brain.

## Author Summary

The cerebral cortex is central to many aspects of cognition and intelligence in humans and other mammals, but our scientific understanding of the computational principles underlying cortical processing is still limited. We might gain insights by considering visual hallucinations, specifically in a pathology known as Charles Bonnet syndrome, where patients suffering from visual impairment experience hallucinatory images that rival the vividness and complexity of normal seeing. Such generation of rich internal imagery could naturally be accounted for by theories that posit that the cortex implements an internal *generative model* of sensory input. Perception then could entail the synthesis of internal explanations that are evaluated by testing whether what they predict is consistent with actual sensory input. Here, we take an approach from artificial intelligence that is based on similar ideas, the deep Boltzmann machine, use it as a model of generative processing in the cortex, and examine various aspects of Charles Bonnet syndrome in computer simulations. In particular, we explain *why* the synthesis of internal explanations, which is so useful for perception, goes astray in the syndrome as neurons overcompensate for the lack of sensory input by increasing spontaneous activity.

## Introduction

Visual hallucinations can offer fascinating insights into the mechanisms underlying perceptual processing and the generation of visual experience in the brain. A pathology known as Charles Bonnet syndrome (CBS) [1]–[4] is of particular interest, for two reasons. First, hallucinations in CBS can be very complex in the sense that they entail vivid, life-like, and elaborate imagery of objects, people, animals, or whole visual scenes. Second, the primary cause of CBS is loss of vision due to eye diseases, with no clear pathology in the brain itself and no necessary impairment to mental health other than the hallucinations. De-afferentation of the visual system and sensory deprivation thus seem to be the important factors in the development of CBS, and comparisons have been made to phantom limb phenomena. Unlike for example in the case of schizophrenia, most often accompanied by auditory hallucinations [5], in CBS there thus does not seem to be a more pervasive malfunction of the cognitive system, but rather some form of over-compensation or maladaptation of the relatively healthy brain to the lack of sensory stimulation.

From a theoretical perspective, there has been an attempt to unify complex visual hallucinations in various pathologies in a single qualitative model [6], but many argue that the underlying causal mechanisms are too varied to do so [7]–[9]. That hallucinations occur in many different circumstances however speaks to them relating to essential aspects of perceptual processing. Thus, theoretical explanations that pose that perception inherently involves some form of active synthesis of internal representations might be well positioned to shed light on the generation of spontaneous imagery in hallucinations, which occur even in CBS where there seems to be little defect in the visual system other than at the input stage. Therefore, two key questions arise here: what do complex hallucinations tell us about perceptual processing in general, and what are the mechanisms triggering CBS in particular?

The purpose of this computational study is hence threefold. First, to gain theoretical insights into important principles of cortical inference by employing the deep Boltzmann machine (DBM) as a model system which is based on such (hypothetical) principles. Second, to examine concrete causal mechanisms for CBS, we model homeostatic regulation of neuronal firing activity, elucidating on various aspects of CBS. Moreover, to examine a potential role of the neuromodulator acetylcholine, we introduce a novel model of its action as mediating the balance of feedforward and feedback processing in the cortical hierarchy. And third, with our results we aim to demonstrate the relevance of Deep Learning approaches such as the DBM as models of cortical processing. A preliminary version of the presented work has been published [10].

### Charles Bonnet syndrome

CBS is characterised by complex recurring visual hallucinations in people who suffer from visual impairment but no other psychological condition or hallucinations in other modalities [1]–[4]. In particular, patients generally gain insight into the unreality of their experiences. The phenomenology of CBS is multifarious, with the nature and content of hallucinatory episodes as well as the conditions favouring their occurrence varying from patient to patient or episode to episode. Common themes are the vividness and richness of detail of the hallucinations, the elaborate content often entailing images of people or animals (though often of a bizarre nature–figures in elaborate costumes, fantastic creatures, extreme colours, etc.), as well as possibly common triggers, such as being in a state of drowsiness and low arousal. Episodes can last from seconds to hours, and hallucinations can reoccur over periods lasting from days to years.

The eponym CBS itself is somewhat ambiguous or even controversial [4], [11]–[13]. Some authors put the emphasis on complex hallucinations in visually impaired but psychologically normal people, where the visual pathology can be anywhere in the visual system from the retina to cortex; others define CBS to be necessarily related to eye diseases only. Similarly, the delineation of the term ‘complex’, and whether CBS should include complex hallucinations only, appears to be not fully clear. On one end are simple or elementary hallucinations consisting of flashes, dots, amorphous shapes, etc., while on the other are fully formed objects or object parts like animals, people, and faces [4], [6]. Somewhere in between are geometric patterns (‘roadmaps’, brickwork, grids, and so forth). Some authors include the latter in CBS [13], [14]. It should be noted that simple hallucinations are actually more common in visually impaired patients than complex ones, with a prevalence of about 50% vs. about 15%, respectively [4]. Both types can occur in individual subjects, possibly with a tendency to progress from simple to complex over time.

For this modelling study, we identify the following key aspects of CBS we aim to capture and elucidate on. First, we take the common definition of *hallucinations* as compelling perceptual experiences in the absence of external stimuli. They are to be contrasted [4], [6] to illusions as misperceptions concerning an actual external stimulus, as well as to mental imagery. Unlike hallucinations, the latter is under complete volitional control, lacks perceptual vividness (it appears to be ‘in the mind's eye’ rather than in the world), and might also have a different neurobiological substrate [13].

Second, in the context of CBS we are interested in hallucinations that are *perceptually rich* in the sense that the experience is similar to that of actual seeing. Presumably, this implies that the representations instantiated in the neuronal activity patterns share significant commonalities in both seeing and hallucinating, though this requires further elaboration.

Third, we consider hallucinations on the *complex* end of the spectrum, i.e. objects, people, and so forth. As we currently lack good generative models of realistic images (biological or otherwise, not counting here of course *purely* generative algorithms from computer graphics that cannot be inverted for inference) the model we employ still relies on relatively simple binary images. However, it attempts to capture at least some aspects of how complex, object-based hallucinations might be created in the brain. For example, the content of complex hallucinations presumably cannot be accounted for by appealing to anatomical organisational properties of lower visual areas, which [14] suggested for simpler hallucinations of geometric patterns in CBS (referring to anatomical “stripes” in V2 etc.). Our model relies on distributed, high-dimensional, hierarchical representations that go beyond local low-level visual features (e.g. V1-like edge detectors). The representations are learnt and reflect structure in sensory data beyond local correlations.

Fourth, with regards to the issue of whether CBS should refer to hallucinations in the context of eye diseases only, our model is meant as a model of processing in the cortical hierarchy, and due to the level of abstraction we only require that *visual input is lost* somewhere at a preceding stage and do not differentiate further. We do however address the distinct roles of cortical areas within the hierarchy.

CBS is a complex phenomenon with manifold symptoms and little data beyond clinical case reports and case series. The aim of our computational model is thus to qualitatively elucidate on possible underlying mechanisms, to demonstrate how several common aspects of CBS could be explained, and to gain some potential insights into the nature of cortical inference.

### Hallucinations and generative models in the brain

The occurrence of complex visual hallucinations in various pathologies [6], [15] as well as the imagery we all experience in dreams show that the brain is capable of synthesising rich, consistent internal perceptual states even in the absence of, or in contradiction to, external stimuli. It seems natural to consider hallucinations in the context of theoretical accounts of perception that attribute an important functional role to the synthesis of internal representations in normal perception, not just in pathological conditions. In particular, one relevant notion is that of perception entailing an ‘analysis by synthesis’, which is an aspect of approaches such as predictive coding or Adaptive Resonance Theory [16]–[23]. The idea is that ambiguous sensory signals inform initial hypotheses about what is in an image in a bottom-up fashion (from low-level image features to high-level concepts, like objects and faces). These hypotheses are then made concrete in a synthesis stage that tests a hypothesis against the image (or low-level representation thereof) by making top-down predictions using a generative process.

In computational neuroscience over the last two decades, this notion of analysis by synthesis and related ones have often been framed in probabilistic or ‘Bayesian’ terms. Generally speaking, Bayesian approaches theoretically describe how inferences about aspects of the environment are to be made from observations under uncertainty (for reviews and introductions, see [24]–[26]). For hallucinations, the relevant aspect of Bayesian models could be that they offer a way of formalising notions of ‘bottom-up’ processing driven by sensory input, and internally generated, ‘top-down’ processing conveying prior expectations and more high-level learnt concepts. An imbalance of, or erroneous interaction between, such ‘bottom-up’ and ‘top-down’ information could underlie hallucinations [27]–[29]. More concretely, the mathematical entities in a Bayesian model or inference algorithm could map to neural mechanisms and processing in the cortex. For example, inference in a hierarchical model could describe hierarchical processing [21]. Top-down processing then would correspond to information flow from higher areas to lower areas, and inference would be implemented via recurrent interactions between cortical regions. Similarly, in the model of Yu and Dayan [27], a concrete biological mechanism is hypothesised to represent the uncertainty of the prior, namely the neuromodulator acetylcholine. The authors thus refer the latter's relevance in some hallucinatory pathologies as evidence, where deficient acetylcholine, corresponding to an over-emphasis of top-down information in Yu and Dayan's account, could lead to hallucinations [6], [15], [30].

As Yu and Dayan [27] state, a shortcoming of concrete Bayesian models such as theirs is that they are often formulated over very simple, low-dimensional, non-hierarchical variables. It is not clear how their treatment of priors and uncertainty translates to models that deal with high-dimensional problems like images in a biologically plausible manner. This is what we need to address if we hope to develop a computational model of CBS, and in this context we will introduce a novel model of the action of acetylcholine in similar spirit to Yu and Dayan's framework.

### Neuronal homeostasis as causal mechanism

While hallucinations in general might relate to an imbalance of bottom-up and top-down in the cortex, the causes behind specifically CBS and the involved mechanisms are poorly understood (for discussion, see [1], [4], [12], [15]). Evidence from CBS and other pathologies suggests that an intact visual association cortex is necessary as well as sufficient for complex visual hallucinations to occur (e.g. [15]). For example, lesions to visual cortex can cause hallucinations, but only if they are localised to earlier areas and do not encompass the higher association cortex. One of the insights emerging from the debate is that the pathology in CBS appears to entail primarily a loss of input at stages prior to association cortex. In contrast, hallucinations accompanying epilepsy, for example, are thought to be caused by an irritative process that directly stimulates association cortices.

How deficient input in CBS leads to the emergence of hallucinations is unclear. Classic psychological theories suggest that the lack of input somehow ‘releases’ or dis-inhibits perceptual representations in visual association cortex. This somewhat vague notion has been made more concrete by taking neuroscientific evidence into account which shows that cortex deafferentiated from input becomes hyper-excitable and generates increased spontaneous activity. As [14] argues (also [12]), changes to neuronal excitability as a consequence of decreased presynaptic input, based on for example synaptic modifications, could thus underlie the emergence of neuronal activity which establishes hallucinatory perception in CBS.

Such adaptive changes of neuronal excitability have been studied extensively over the last two decades in experimental and theoretical work on *homeostatic* plasticity (see [31] for review; also [32], [33]). Rather than deeming them artifacts or epiphenomena, such changes have been attributed important physiological functions, allowing neurons to self-regulate their excitability to keep their firing rate around a fixed set-point. Homeostatic regulation is thought to stabilise activity in neuronal populations and to keep firing within the neurons' dynamic range, compensating for ongoing changes to neuronal input either due to Hebbian learning, or due to developmental alterations of the number of synapses, connectivity patterns, etc.

A neuron might track its current activity level by measuring its internal calcium levels, and several cellular mechanisms have been identified that could then implement its homeostatic adaptation. Among them is ‘synaptic scaling’, a change to synaptic efficacy that is thought to affect all synapses in a neuron together, keeping their relative strengths intact. Alternatively, the intrinsic excitability of a neuron can be regulated by changing the distribution of ion channels in the membrane. Both mechanisms have been observed experimentally, dynamically regulating neuronal firing rate over a time-span from hours to days [34] in compensation for external manipulations to activity levels–in particular, in response to an activity decrease caused by sensory deprivation.

Hence, with visual input degraded due to eye disease or other defects in the visual pathways, homeostatic *over* compensation is a strong contender to be the neuronal cause underlying the emergence of hallucinations in CBS. This is the mechanism we explore in our computational model.

## Model

To address CBS, we need to work towards computational models that can capture its key properties as identified earlier. Such a model should be able to internally synthesise rich representations of image content, such as objects, even in the absence of (corresponding) sensory input. We now briefly describe the deep Boltzmann machine (DBM). This being the first work that applies DBMs as models of cortical processing, we discuss its interpretation as a biological model. We also specify the parameters used in the simulation experiments. For a more extensive explanation and discussion of all aspects of the DBM framework brought up in this section, see [35].

### Deep Boltzmann machines

DBMs are probabilistic, generative neural networks that learn to represent and generate data in an unsupervised fashion. They consist of several layers of neuronal units arranged in a hierarchy. The units fire stochastically, inducing a probability distribution over the network state, parametrised by the weight (and bias) parameters, i.e. the connection strengths between units. DBMs were introduced recently in machine learning by Salakhutdinov and Hinton [36]. While Deep Learning approaches such as the DBM are often taken to be inspired by the brain [37], the relevance of the DBM as a concrete model of processing in the brain has not been explored so far. We argue that DBMs are valuable as models of (hypothetical) aspects of cortical processing, as the computational principles they are based on could play an important role in cortical learning and processing as well.

A DBM is a special case of a general Boltzmann machine (BM) by virtue of its specific architecture. BMs themselves were developed in the nineteen eighties [38]. The reason that DBMs have enjoyed recent interest in machine learning is not that the basic underlying model formulation of a BM has changed; rather, recent developments in learning algorithms have made it possible to effectively train these models, taking advantage of their ‘deep’ structure to overcome earlier problems that made the application of BMs difficult.

**Figure 1:**
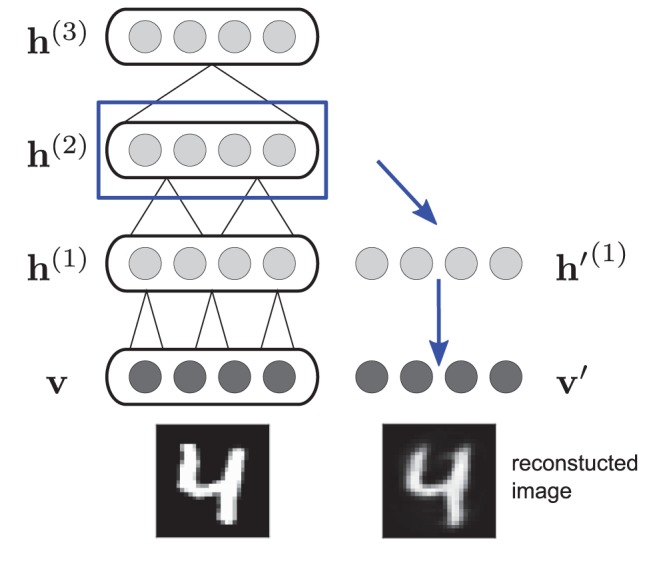
Decoding the internal state. During perception, the states of any hidden layer are decoded using a copy of the DBM as a decoder. Starting from the hidden states of the layer in question, a single deterministic top-down pass is performed to obtain a reconstructed image.

Concretely, a DBM consists of 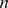 layers of neurons (e.g. Figure 1). Usually, the states of the neurons are taken to be binary, 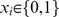, indicating whether a unit is ‘on’ or ‘off’, but other choices are possible, such as continuous-valued, rectified linear units [39]. The states of each layer are written as vectors, denoted by 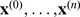 (together denoted by 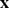). Units 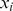 and 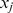 in adjacent layers are connected by symmetric connections with connection weight 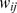, the latter modelling synaptic strength. For each adjacent pair of layers layers 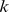 and 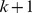, the weights can be combined into a weight matrix 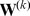. Each unit also has a bias parameter 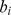 that determines its basic activation probability by functioning as a baseline input. In a default DBM, there are no lateral connections between units within a layer.

The first layer constitutes the *visible* units, i.e. they represent the input data, such as the pixels of images. The higher layers contain *hidden* units that are not given by the data. Rather, their states form a distributed representation of the input data, the meaning of which is assumed in learning. There, the parameters (weights and biases) are adjusted to learn a good internal model of the sensory input, in a sense to be described below.

Each unit 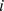 receives input 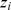 from the other units it is connected to via the weights (plus the bias),
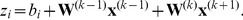
(1)This input determines the probability for the unit to switch on. For binary units, it is computed using a sigmoid (logistic) activation function:
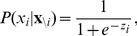
(2)where 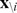 denotes all unit states other than 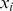. 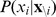 is also called the activation (probability) of unit 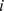.

If the DBM is run over a long enough time, by stochastically activating its units, then the probability to find the network in any state 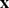 asymptotically converges to an equilibrium distribution. In analogy to a system described by (classical) statistical thermodynamics (specifically, the Boltzmann machine corresponds to an Ising model), this distribution is given by the system's Boltzmann distribution (assuming a temperature of 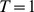),
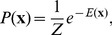
(3)where 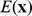 is called the energy of the system and is defined as
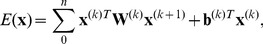
(4)and 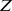 is the normalisation constant.

### DBMs as neural networks and probabilistic models

DBMs can be understood from two perspectives. The first is to view DBMs as neural networks, simple models of neuronal processing on a comparable level of abstraction and idealisation as other connectionist-style networks used in machine learning and computational cognitive models. In particular, BMs in general can be seen as a generalisation of the Hopfield network [40], [41], which has been used as a basic model of memory storage and recall in neuronal cell assemblies [42]. BMs differ from Hopfield networks in two fundamental respects. First, in the latter, the activation rule is deterministic. Initialised in some state, a Hopfield network will converge to a state that forms a local minimum in the energy ‘landscape’. Learning aims to sets the weights such that this state corresponds to one of the input patterns to be memorised. BMs on the other hand explore the energy landscape stochastically, potentially traversing several minima in the process. The second difference is that Hopfield networks do not have hidden units. Hidden units enable BMs to learn aspects of the data that are not defined by pairwise correlations. Moreover, rather than just capturing correlations between visible units (e.g. pixels in an image) in the weights between them, hidden units can represent specific patterns or features in the visible units, and explicitly signal their presence or absence by virtue of their state. Thus, rather than just memorising patterns, BMs can learn internal representations of sensory data.

The fact that DBMs compute distributed hidden representations in several non-linear processing stages also relates them to feedforward neural networks. However, whereas the latter are usually trained by providing desired output values (i.e., in a supervised fashion), such as image labels, and tuning the weights with the backpropagation algorithm [43], DBMs learn without supervision, attempting to find an internal model from which the input data can be generated.

The second perspective on BMs (and DBMs), perhaps more in line with modern machine learning approaches, views them as probabilistic graphical models of data. In this context, a BM is an instance of a Markov random field, which is a probabilistic graphical model whose independence relationships are captured by an undirected graph (e.g. [44]). Rather than introducing BMs on the basis of the stochastic activation rule, one can instead start from the Boltzmann distribution, Eq. 3, as a definition of the model via its joint distribution over the random variables 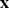, and then derive the activation probability (Eq. 2) for each unit simply as conditional probability. ‘Running’ the BM stochastically then produces samples from the joint distribution. In fact, iteratively sampling each unit's state according to its conditional probability implements Gibbs sampling, a Markov chain Monte Carlo (MCMC) method (see e.g. [45]). MCMC and similar sampling-based methods have been suggested to relate to cortical probabilistic inference [26], [46]–[51], and it is focus of our work on modelling bistable perception within the DBM framework [35], [52].

### DBMs as models of cortical inference

We argue that the DBM is promising as a model of hallucinations, and other aspects of a hypothetical generative model in the cortex, because it implements a generative model that learns to synthesise representations of sensory data. A DBM can be seen as an instance of a hierarchical probabilistic model, and thus could capture the intuition of bottom-up and top-down processing in the cortex reflecting the interaction between sensory information and internal priors. An imbalance of such processing then can be seen as a cause for hallucinations to emerge. At the same time, the DBM is also a simple neural network, thus enabling us to explore concrete neural mechanisms possibly underlying CBS. Because the DBM does not just memorise given input patterns like the related Hopfield network (which itself has been used to model hallucinatory ‘memories’ in schizophrenia [53]), but rather learns *internal* representations of input images, it is a more concrete model of *perception* rather than just memory. The ‘deep’ organisation of the DBM into hierarchical layers as well as the image based representations will allow us to make concrete connections to the visual cortex.

The DBM being a generative probabilistic model of sensory data, the act of perception corresponds to inferring the hidden or latent variables that are consistent with and could have generated the observed input. We make a clarification here in light of a current debate concerned with the merit and meaning of approaches to cognition termed ‘Bayesian’ [54]–[58]. The approaches in focus there are characterised as rational, optimal, or ideal observer models. They are meant to describe specific perceptual inference problems, capturing what can *in principle* be inferred about a specified property of the environment from sensory data. In contrast, in case of the DBM model, the probabilistic framework is used to develop a (component) solution to perceptual tasks, perhaps capturing aspects of processing in the brain, but this solution does not need to be ‘optimal’ in any sense. In particular, the hidden variables in the DBM do not have by design *a priori* meaning assigned to them in terms of the environment, but rather attain any meaning due to whatever useful representations are discovered in learning. Thus models like the DBM differ conceptually from ideal observer models [35], but these different approaches can still be related to each other as they are based on the same theoretical language of probabilistic inference.

Seen as a model of aspects of cortical processing, the DBM is a rough idealisation, but comparable in that regard to other related modelling approaches [35]. As we will show, the DBM does capture several hypothetical aspects of cortical processing relevant for explaining CBS.

### Learning

Developing flexible models that can learn useful representations of many kinds of sensory data is one of the key motivations behind Deep Learning approaches such as the DBM. Such versatile learning could also be what makes the cortex so flexible and powerful across many sensory modalities. The learning algorithms for BMs, and DBMs in particular, are themselves not focus of our work on hallucinations here, but we summarise the key points below (see Supplementary Text S1 and [35] for further comments on the biological relevance and plausibility).

Taking the probabilistic model interpretation of a BM, learning can be derived as likelihood optimisation of the model parameters given some sensory training data. Notably, the resulting iterative update rule for the weights of the model involves only local Hebbian learning, and an alternation between two phases where the BM either performs inference over some input or freely generates from its internal model (this second phase could possibly offer a normative explanations for dreams [59]).

There are three key aspects to why BM-based models have found renewed interest in machine learning over the recent years. First, the focus turned to BMs with simplified connectivity, in particular the Restricted BM (RBM), where neither visible units nor hidden units have connections amongst their own type (a RBM is equivalent to a 2-layer DBM). Second, making use of the simplified inference in such models, more effective approximate learning algorithms were developed, such as the Contrastive Divergence algorithm [60]. Third, RBMs were used as building blocks to train deeper, multi-layer architectures such as the DBM. Treating each pair of adjacent layers as its own RBM, the DBM is initially trained one subsequent layer at a time, with each hidden layer learning to generate the unit states in the respective layer below. Once the whole DBM is composed, further training can then be performed on the whole model.

The biological relevance of deep RBM-based models such as the DBM has been examined by matching the learnt neuronal receptive fields to those of neurons in the visual cortex [61], [62]. Our study here is the first to explore the potential of the DBM as a biological model beyond receptive field properties.

### Decoding the internal state

To model perceptual phenomena with the DBM, we feed sensory input to the model by clamping the visible layer to images, sampling the hidden layers, and then analyse what is represented in the states of the hidden layers during inference. In the case of hallucinations we are in particular concerned with perceptual content that is *not* matching the actual visual input. To decode the hidden states in terms of the sensory data they represent, we can make use of the generative nature of the DBM and ask what images would be generated from the hidden states in question. We thus take another DBM instance with the same parameters as the DBM used to model perceptual inference to implement a decoder. For any set of hidden states, the decoder is applied to obtain reconstructed input images for each hidden layer independently (Figure 1).

Specifically, given the states of any hidden layer 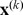, 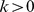, at any point during perceptual inference, we set the respective hidden layer in the decoder DBM to the states to be decoded, and then perform a single deterministic top-down pass starting from there: the activations in each subsequent lower layer are computed using only the layer above as input (propagating 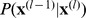), until a reconstructed image is obtained in the visible layer of the decoder (taking probabilities as grey-scale values). The weights in the decoder are doubled to compensate for the lack of bottom-up input (analogously to the bottom-up initialisation used in [36]). Possible alternatives to this decoding procedure are discussed in [35].

### Homeostasis in a DBM

We model CBS as resulting from homeostatic regulation of neuronal excitability in response to degrading visual input. We use DBMs that have learnt to represent images, having trained them on either of two simple data sets. We then simulate the visual impairment by using empty or corrupted input instead of the original data, and have the model perform inference over them. The change in sensory input could lead to changes in the activation levels of the model's neuronal units. To model homeostatic mechanisms, we allow the neurons to adapt their excitability in response.

**Figure 2:**
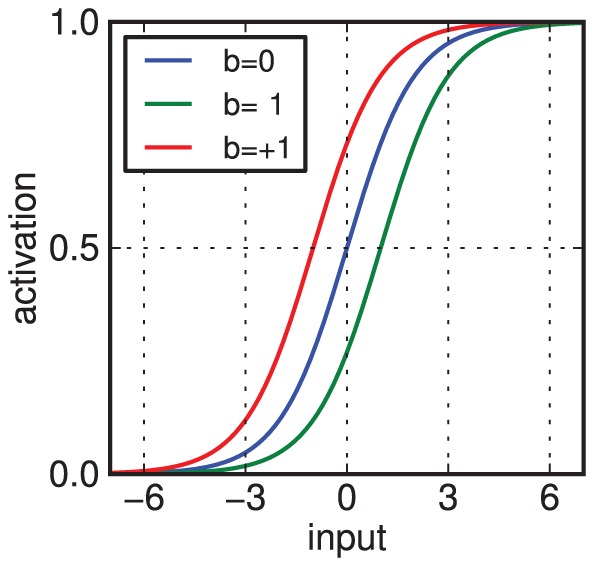
Homeostatic excitability shift. The activation probability (given by the logistic function) of a neuron shifts depending on the value of the bias parameter 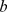.

As discussed earlier, homeostatic plasticity can be described as a neuron adapting its excitability to match its current average firing rate (as measured over hours or days) to a fixed set-point [33], and there are several cellular and synaptic processes making this possible. Here, for simplicity we model a single basic mechanism, namely an iterative adaptation of each neuron's intrinsic excitability. With target activity 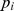 and current average activity 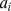, neuron 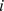 in the DBM should become either more or less excitable according to the difference 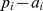. Its bias parameter 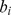 is thus iteratively incremented by
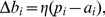
(5)where 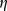 is a constant parametrising the rate of adaptation. Such an adaptation of the bias has the effect of shifting the activation function of the unit, i.e. the probability for it to switch on, rendering it more or less excitable for a given amount of input (Figure 2; cf. Figure 3a in [31] on homeostatic plasticity).

**Figure 3:**
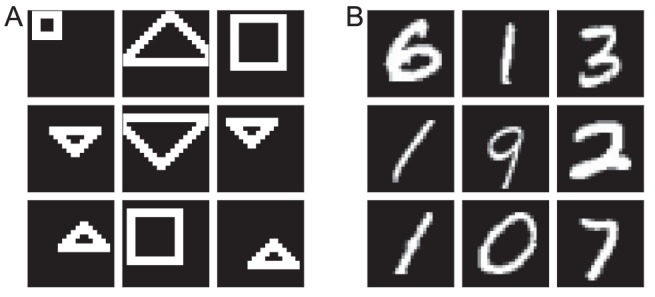
Examples from the training data sets. See main text for details. (A) A custom data set of simple shapes at various positions. (B) The MNIST data set of handwritten digits, a standard benchmark in machine learning.

To define the target activity 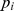 for each neuron, we simply take the average activity of a unit during inference over the training data (after training) as the normal, ‘healthy’ level of activity for the representations learnt. An alternative would be to use the homeostatic mechanism during training itself, specifying a target activity level for the neurons. This corresponds to a regularisation that has been used in machine learning e.g. to enforce sparsity in the representations [61], [63] (weight decay during training [64] could be seen as another type of homeostatic mechanism akin to synaptic scaling). We report here results without using this mechanism in training itself, but we obtained similar results when trying the latter. Thus, what mattered here is only that the activity levels assumed during training were restored, regardless of whether these levels were originally confined to a certain regime.

### Simulation setup

We used two training data sets to explore different aspects of CBS (Figure 3). The first is a custom set of binary images containing toy shapes of various sizes at various positions. This shapes data set allowed us to examine issues related to the localisation of visual impairment, and due to its simplicity the perceptual content of the corresponding hallucinations is straightforward to analyse by directly comparing it to training images. The second data set is MNIST, which contains images of handwritten digits and is a standard benchmark used in machine learning. The advantage of MNIST is that it contains objects that, if still simple, arguably have some more interesting structure. With such kinds of data it has been shown that DBMs can learn representations that generalise to unseen instances of the data, not just in terms of classification performance but also in terms of the data they generate themselves [65]. This in particular demonstrates that learning does not simply correspond to memorising training images.

For both data sets, the employed DBMs had three layers of hidden units. The weights between layers were restricted to implement localised receptive fields so that each unit was connected only to a patch of adjacent units in the respective layer below. Receptive fields in the highest hidden layer were global. The biases of the units were initialised to negative values before training to encourage sparse representations. In particular, this lead to a breaking of symmetry between on and off states: by encouraging units to be off most of the time, they learn representations where they signal the presence of specific content in an image by switching on [35], [66]. Input degradation (which models visual impairment) then generally had the effect of decreasing neuronal activity, and in consequence homeostatic regulation would have to recover firing rates by increasing the excitability of the units. This matches the findings that cortical neurons become ‘hyper-excitable’ under sensory deprivation (as reviewed e.g. by [14]). Other than the sign of the activity changes, overall results as reported in this study did not however depend on representations being sparse.

For MNIST, the visible layer had 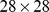 units corresponding to the size of the images in pixels, and 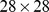, 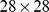, and 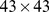 units in the three hidden layers, from lowest to highest, respectively. Receptive field sizes were 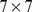, 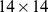, and 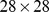. The model was trained layer-wise for 30 epochs (i.e. iterations through the training data) in each layer, using 5-step Persistent Contrastive Divergence (Supplementary Text S1). The training set contained 60,000 images, 6,000 per digit category (0 to 9). For the shapes data set, the visible layer had 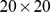 units and the hidden layers 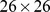 units each, with receptive field sizes 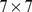, 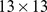, 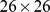. Here, layer-wise training consisted of 30 epochs of 1-step Contrastive Divergence (Supplementary Text S1). The training set again had 60,000 images in total, from six categories (squares, triangles in two orientations, all in two different sizes). It should be noted that, due the limited variability in the shapes data set, all possible image instances were covered by the training set. Hence, only the MNIST data set is suitable to test the generalisation performance of the model. Lastly, for neither MNIST nor the shapes data set were the models trained further after the layer-wise pre-training. See Supplementary Text S2 for further details on the training parameters used.

To measure the preferred activity 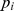 for each hidden neuron, we averaged its activation over all training data (after learning), with one trial per input image consisting of 50 sampling cycles. Here and elsewhere, the hidden states were generally initialised to zero at the start of a trial. Similarly, to measure the current average activation 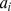 during homeostatic adaptation, activities were measured over 50 cycles in 100 trials per iteration. Depending on the experiment in question, the visible units were set to a different image for each trial or remained blank (when modelling complete blindness). The adaptation rate 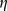 was set to 0.1 and 0.04 for models trained on shapes or MNIST, respectively, with a lower rate for MNIST as the model was found to effectively adapt faster for this data set. For the overall results, the precise value of the rate did not matter.

To analyse the perceptual state of the model, we decoded the states of the hidden layers as described earlier, obtaining a reconstructed image for each layer at each sampling step. To evaluate the internal representations w.r.t. their possibly hallucinatory content, we analysed whether the decoded images corresponded to the kind of objects the models had learnt about in training, using the topmost hidden layer's states after 50 sampling cycles for quantitative analysis. For the shapes data set, we employed a simple template matching procedure, matching the image to the shape templates used in training by convolving the former with the latter (each image had its mean subtracted and was then 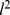 normalised). The maximum value of the resulting 2D vector was taken as quantitative measure for the correspondence, termed the ‘hallucination quality’, where a perfect match corresponded to a hallucination quality of 1.

For the more varied MNIST data set, there are no fixed templates, nor do generated images necessarily match instances from the training set (which is the point of having a model that can generalise, as mentioned above). To obtain a measure of hallucination quality, we classified the decoded image as belonging to any of the digit categories, using the confidence of the classifier as a measure of the image's quality. Specifically, we used an instance of the DBM model itself (not affected by homeostasis) with a classification unit attached (see e.g. [67]). Taking the maximum of the posterior over the digit categories again yielded a measure with maximum value 1. Inspecting the generated image and resulting posterior values, we also confirmed that for images that did not look like well-defined MNIST digits, classification scores computed in this manner tended to be lower. It should be noted that the aim of our work was not achieving high classification performance, hence we did not train the full model, fine-tune the hyper-parameters, nor necessarily implement classification in an ideal fashion. Classification is merely used to analyse the quality of the internal representations. The reported error rate for MNIST (7%) is hence higher than the state of the art, the latter being around 1% for this type of model (e.g. [68]).

## Results

The hypothesis we explored is that homeostatic regulation of neuronal firing rate in response to sensory deprivation underlies the emergence of hallucinations in CBS. The possibility for synthesis of internal representations is explained by the cortex implementing a generative model of sensory input. As a first step, we aimed to demonstrate that the homeostasis mechanism as implemented in the model can actually be beneficial in this context.

### Robust analysis by synthesis due to homeostasis

**Figure 4:**
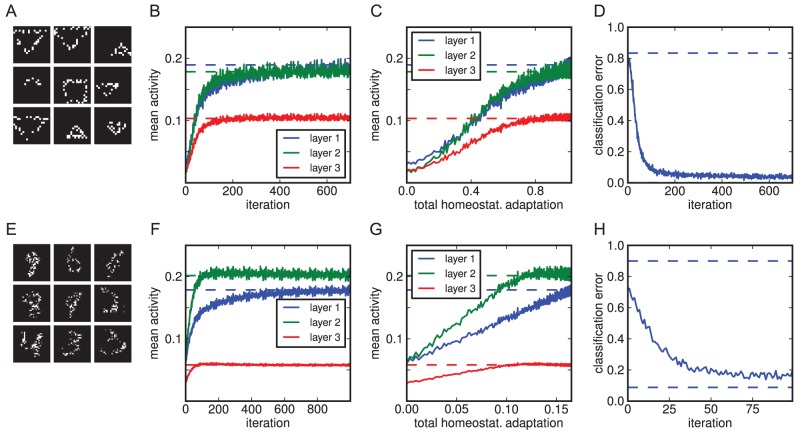
Homeostatic adaptation restores activity levels and internal representations when input is corrupted. (A) Examples of corrupted images for the shapes data set. (B) Average activity levels in each of the three hidden layers over the course of homeostatic adaptation. Activity levels are plotted against the number of iterations so far. Dashed lines correspond to normal activity levels for each layer with uncorrupted input. Activities initially dropped profoundly as input was corrupted, but then recovered as the neurons adapted. (C) As in B, but plotted against the total homeostatic adaptation in the neuronal bias parameters (absolute differences between current bias values and initial values, averaged over all units). (D) Classification error using the internal representations to classify the corrupted input (see text for details). Top dashed line is chance, bottom one is performance on uncorrupted input (here, for the simple shapes data set, the error is very close to zero, hence the corresponding line is drawn on top of the x-axis). Over the course of adaptation, internal representations are restored as well, allowing for classification performance close to its original level. (E–H) Analogous to A–D, but for a model doing inference over corrupted MNIST images.

In the following, we show how homeostatic adaptation could be helpful in particular for a model that implements perceptual inference by synthesising internal representations, by making the learnt representations robust against exactly the sort of visual degradation that ultimately causes CBS. To this end, we had the model (trained on either the shapes or MNIST data sets) perform inference over heavily corrupted versions of the images (Figures 4A and 4E). The latter were created by taking images from the data sets (digit instances not seen in training in the case of MNIST) and setting 65% of the pixels to black.

Degrading the input in this manner lead to profound activity changes in the neurons, which the model was then allowed to compensate for by employing homeostatic adaptation. Figure 4 shows how activity levels changed under input degradation and subsequent adaptation, plotted either against the number of preceding iterations or the total shift of the bias parameter so far (averaged over all units). For all three hidden layers, initial activities were lower when compared to normal levels. Homeostatic adaptation then led to a gradual restoration to the original values.

Importantly, this recuperation of activity levels corresponded to a restored capability of the model's internal representations to capture the underlying objects in the images. We decoded the hidden states of the top layer and classified the resulting reconstructed images using a classifier trained on the original data sets. Input degradation initially lead to a sharp drop in performance in classifying the corrupted images (Figure 4D and 4H). However, homeostatic adaptation lead to a significant improvement of classification, reaching a performance that was close to the one achieved on the decoded representations inferred from uncorrupted images.

Hence, the homeostatic mechanism as defined by Eq. 5 can be sufficient to restore the representations inferred over sensory input as to be suitable for classification. This is despite the fact that it only attempts to match the average activations, i.e. first order statistics of the inferred posteriors averaged over all input images, rather than the full distribution learnt in training, and only does so by adapting the bias parameters. Thus, homeostatic adaptation could offer a simple local neuronal mechanism that serves to make learnt representations robust for example against degradation of sensory input. It does not rely on further learning (in the sense of parameter changes that incorporate incoming sensory data), intricate synaptic changes, or network wide measurements. Rather, each neuron only needs to remember its average activity level and regulate its intrinsic excitability accordingly. However, as we will see in the following, this stabilisation of perceptual representations can be detrimental, ultimately decoupling internal representations from a further degraded sensory input, causing hallucinations.

### Emergence of hallucinations

**Figure 5:**
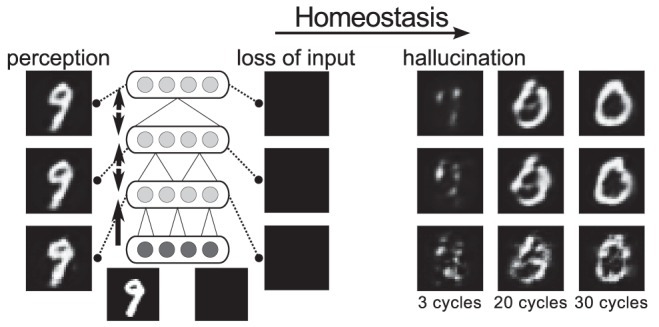
Overview of the basic CBS experiment. A model has been trained on simple images (here, MNIST digits). Initially, decoded internal representations correspond to what is given as input in the visible layer. To model visual impairment or blindness, sensory input is then removed, eliciting internal representations devoid of content. Subsequent homeostatic adaptation of neuronal excitability leads to spontaneous hallucinatory representations emerging (right-hand side images are decoded from the hidden layers, receiving no sensory input, 3, 20, or 30 sampling cycles after initialisation).

To model more profound visual impairment or blindness, we then repeated the above experiment but with the visible units permanently clamped to completely empty input. As before, the model had initially been trained on images from either of the two data sets. With the model now performing inference over empty input, homeostatic adaptation was again allowed to take place. Any emergence of meaningful internal representations in the absence of input would correspond to hallucinations. See Figure 5 for an overview of the CBS experiment.

Before presenting the results, we should briefly comment on how the binary input images are to be interpreted so that presenting a blank image corresponds to ‘taking the input away’, i.e. blindness. After all, seeing a black image is not the same as not seeing altogether. Rather, the binary images are here to be understood as proxies of images already encoded in neuronal activity at an early stage of visual processing (e.g. primary visual cortex). We here do not model this earlier encoding for simplicity, but will consider equivalent cases later in experiments where we model loss of vision in higher stages of the hierarchy.

**Figure 6:**
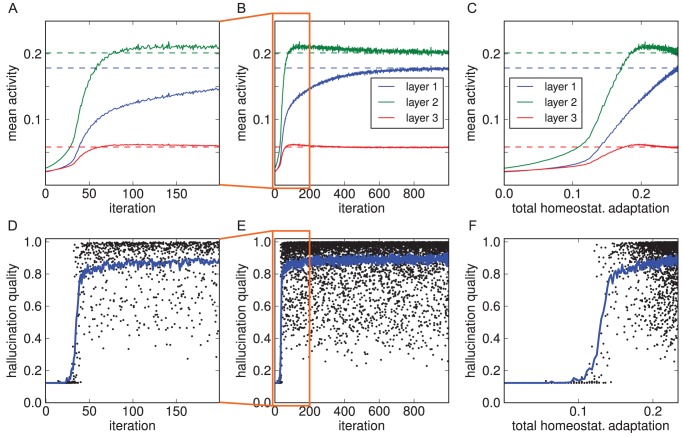
Emergence of hallucinations due to homeostatic adaptation. The model was trained on the MNIST data set (results for the shapes data set were equivalent, Supplementary Figure S1). (A–C) With empty images as input, activity levels dropped in all three hidden layers and then recovered over the course of homeostatic adaptation (original levels as dashed lines; see Figure 4 for explanation of x-axes). (D–F) Quality of hallucinations (i.e. how well decoded internal representations matched the learnt images). Each dot represents the decoded internal state after the 50 sampling cycles constituting a trial (5 out of 100 trials per iteration are plotted). Blue curve denotes mean quality over 100 trials in that iteration. After an initial period of silence, hallucinations emerged abruptly, quickly rising in quality. The emergence of hallucinatory representations coincided with a more rapid recovery of activity levels.

Figures 6A–C show the activity changes resulting from visual impairment and subsequent adaptation for a model trained on MNIST (results for the shapes set were equivalent, Supplementary Figure S1). Again we found an initial drop of activity that was subsequently fully compensated for, at least on average over each hidden layer, by the shift of the intrinsic excitability of the neurons.

What was the nature of the internal representations that allowed for a restoration of activity levels? After all, the purely local adaptation of each neuron might have recovered individual preferred firing rates on the basis of noisy firing or other activation patterns that bore no meaningful representations according to what the model had learnt about initially. Instead, when we decoded the hidden states of the model we found that the represented content after adaptation corresponded to the kind of images seen in training, whereas prior to adaptation, decoded images matched the empty input.

To quantify this, we measured hallucination quality (as defined in the Model section) over the course of homeostatic adaptation. In Figures 6D–F, each dot represents the quality of the image decoded from the topmost hidden states at the end of the 50 sampling cycles in a trial. It becomes apparent that hallucinations started to emerge only after an initial period of silence, even as excitability was already adapting. This is consistent with cases reported in CBS where loss of vision was abrupt [4]. The reported duration of this latent period, ranging from hours to days, in turn matches well the time scale over which homeostatic adaptation takes place [34].

**Figure 7:**
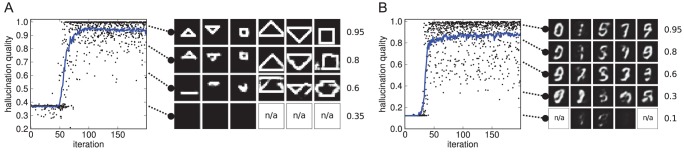
Example decoded hallucinations. Examples (right-hand side) are shown with corresponding scatter plots for reference (left-hand side; from Figures S1D and 6D). (A) for the model trained on shapes, displayed are examples from the six shape categories (columns, as categorised by matching to the shape templates), for four different qualities (rows, with quality values listed on the right-hand side; images were of that quality or within +0.05 thereof). For entries marked ‘n/a’ there was no hallucination of that type and quality (note that the categories are not really meaningful for lowest quality images anyway). (B) Similar to A, but for the model trained on MNIST. Examples shown were classified as belonging to digit categories 0, 1, 5, 7, and 9 (columns), for five different qualities (rows, annotation as in A). MNIST hallucinations of lower quality often looked like less well-defined digits or mixtures of different digit classes, or they would deviate from the categories in the training set in more subtle ways. Human judgement of quality and class could deviate from the classifier's results in such cases.

In terms of quality, high-quality hallucinations were found soon after the point when hallucinations emerged (see Figure 7 for example decoded hallucinations). That point also marked a profound increase in the rate of activity changes. This shows that the emergence of stable internal representations is not just a epiphenomenon of underlying activity changes, but rather itself plays a key role in the system recovering normal activity levels.

Throughout the course of adaptation, we found there to be a mix of hallucinations of various qualities. Lower quality images could correspond to temporary states as the model transitioned from one relatively stable state to another. Note that within any one trial, the model never converges to a fixed internal state, as it keeps stochastically sampling from the posterior. We did observe a tendency to stay within one category of object (e.g. a specific class of digit) towards the end of a trial, but this is simply a general property of such models not specific to the hallucinations (we address this issue in [35], [52]). Similarly, hallucinations could come from various object categories (among the digit or shape classes) for an individual instance of the model. This matches reports from CBS patients, which indicate there can be a variety of hallucinatory content that varies from episode to episode for an individual subject [2], [4]. It is thus important that the model could produce varied representations rather than just a few degenerate states.

### Sensory deprivation due to noise or impoverished input

The emergence of hallucinations in the model does not require complete lack of input. We obtained similar results when performing the homeostasis experiment with images containing, for example, some noise (10% white pixels on black background randomly sampled for each image). In that case, fewer iterations and less homeostatic adaptation were needed to trigger hallucinations (Supplementary Figure S2). Hence, the nature of visual impairment can have an impact on when or whether hallucinations are occurring. This could also offer one possible explanation for why there might be a tendency for hallucinations in CBS to cease once vision is lost completely [4]. If one assumes that there are limits to how much neurons can adapt their excitability, then some remaining input, even if it is just essentially noise, might be necessary to drive cortical neurons sufficiently. On the other hand, an alternative explanation for a cessation of hallucinations might be long-term cortical reorganisation or learning (see Discussion).

Still, one potential problem with our implementation of sensory degradation so far, be it with empty input or noise, could be that it corresponds to a rather extensive damage to the visual system. Perhaps one would be inclined to interpret such input degradation as a model of complete blindness rather than a more graded visual impairment (or one that is more spatially restricted, see the next section), where in the latter case there might be some structure in the sensory data left. Moreover, in all experiments simulated so far, the emergence of hallucinations occurred due to homeostatic adaptation that compensated for a rather massive drop in activation levels caused by the lack of input. However, if the introduced homeostatic mechanism is truly effective at stabilising the *distribution* of learnt internal representations, one could expect that the system could be prone to hallucinate under much more general conditions than just lack of input: as long as the ongoing input does not evoke a wide *variety* of learnt percepts, those groups of neurons that participate in representing the lacking percepts might compensate by increasing their excitability, possibly causing corresponding hallucinations.

To address these issues, we aimed to test whether hallucinations were exclusively a consequence of compensation for overall lack of input and resulting activity decreases, or whether they could still emerge with structured input that was however highly impoverished in its variety. To this end, we simulated the homeostatic adaptation for the shapes and MNIST models, with the visible layer clamped to only a *single* fixed image from the respective data sets over the course of the whole experiment. To clarify, as before, this models slow neuronal changes over the course of perhaps days or longer, rather than fast neuronal adaptation during ongoing perception, with neuronal parameters being fixed during trials and only updated gradually between them.

**Figure 8:**
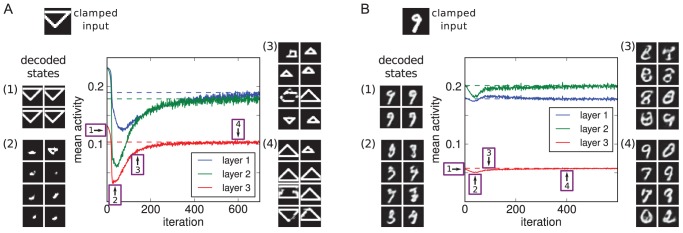
Modelling sensory deprivation due to impoverished input variety. (A) Model trained on shapes. (B) Model trained on MNIST. The input layers of the models were clamped to a single image from the respective data sets throughout the course of homeostatic adaptation. Plotted are resulting activity changes and example decoded internal states. Initially, decoded images (1) corresponded to the input. As neurons adapted and the internal percepts deviated from the true input, global activities dropped (2), then recovered driven by hallucinatory percepts (3 and 4). For particularly the MNIST model, we also observed a more gradual improvement in hallucination quality (compare B3 to B4).

Results are displayed in Figure 8, depicting activity changes over the three hidden layers and examples of decoded internal representations at various stages. We found that hallucinations did indeed develop: initially, the decoded internal states faithfully represented the image in the sensory input. However, as the neurons adapted over time to compensate for the impoverished input, the internal representations entailed objects not actually in the image, effectively decoupling perception from sensory input.

This result clarifies that the action of the homeostatic mechanism can be much more specific than just recovering overall activity levels. Indeed, for the fixed input images used, initial global activity levels when doing inference were actually at or above average, for the MNIST model and shapes model, respectively (as is shown in the figures). The homeostatic adaptation however acts locally for each neuron. With fixed input, one sub-population of neurons, whose activation distributedly codes for that input, will be highly active, while other groups of neurons are less active than average. Adaptation of neuronal excitability can then continuously shift the balance, even if activity averages across a layer remain similar (we examined a related functional role of neuronal adaptation on shorter time scales in [35], [52]).

As can be observed in the figures, there was an initial drop of global activity levels, especially for the shapes model. Based on the decoded representations at that point, we suggest that this results primarily from the neuronal population that represents the initial, veridical percept decreasing excitability. Then, as other neurons increase their respective excitabilities, alternative, hallucinatory internal representations take over, leading to a stabilisation of global activity levels.

The degree of decoupling of the internal percepts from the sensory input was striking. It appeared to be surprisingly robust, overcoming not just a lack of input but even contradictory input. In the case of the shapes in particular, the hallucinated objects do not even necessarily share parts with the true input. It should be recalled that the homeostatic mechanism merely adapts the local biases, and thus does not at all change the connection strengths between units or layers. Indeed, we could show that the flow of information from sensory input to the higher layers was not completely prohibited in the model after homeostatic adaptation. Running a model that currently displayed hallucinatory representations as if decoupled from input, we modestly increased the impact of feedforward processing, using a mechanism meant to model the action of acetylcholine (to be introduced below). The internal representation then reliably realigned to the actual input image.

### Localised and miniature hallucinations from localised impairment

**Figure 9:**
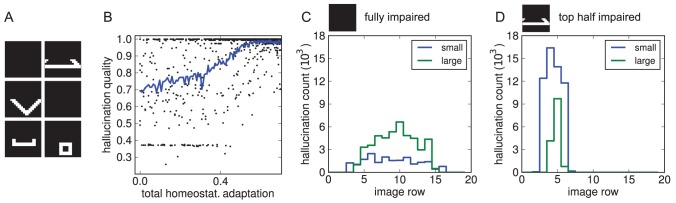
Hallucinations with visual impairment restricted to the top half of the input. (A) Example images. (B) Hallucination qualities during adaptation. Note that many of the corresponding decoded internal representations were not actually hallucinations, but rather matched shapes that were in the unimpaired half of the visual input. In particular, in the early phase of adaptation there are two clusters at low and high quality values. These correspond to void internal representations or veridical ones when shapes happened to lie completely in the impaired or healthy halves, respectively. The former then were gradually replaced with emerging hallucinations. (C) Distribution of hallucinated small and large shape categories across the image in the model with fully impaired input. Only hallucinations with quality greater than 0.85 were counted here. (D) As C, but for the model that underwent adaptation with only the top half damaged (displayed data then taken with fully blank images as input as to not be influenced by actual objects in the healthy region). Now, hallucinations were localised to the impaired region and favoured smaller shapes, which would ‘fit’ within that region.

Visual impairment leading to CBS can also be constrained to specific parts of the visual field. Although reports are conflicting [4], for some patients at least hallucinations tend to be localised to these regions. We tested whether we could reproduce this finding using the model trained on the shapes data set, in which the objects are distributed across various image positions. We simulated a more localised impairment by repeating the homeostasis experiment while blanking only half of the images (for example the top half, Figure 9A). As before, the neurons' activities dropped initially and then recovered during adaptation as hallucinations emerged (Figure 9B).

In the original homeostasis experiment, where visual impairment involved the whole visible layer, hallucinated objects were distributed across the whole visual field (Figure 9C). However, when the model where only half of the images had been blanked was tested (on blank images), hallucinated objects were restricted to the image region that had been lesioned (Figure 9D). Excitability changes due to homeostatic adaption are thus specific enough in the network to have topographic properties.

Another occasional phenomenon in CBS is that hallucinated objects appear to be “Lilliputian” or miniaturised. It has been suggested that this can be explained as resulting from a mismatch of hallucinated content and context, where hallucinations appear against real visual background that happens to be too close in relation to the size of the hallucinated objects [11]. On the basis of our simulation results, we tentatively make another prediction: if there is a propensity for hallucinatory content to consist of meaningful wholes, such as full objects or faces, then in patients where hallucinations are restricted to impaired regions of the visual field there should be a correlation between object size and the spatial extend of visual impairment. To see this in our model, consider that in our shapes data set, objects could come either in small or large versions. For models with full loss of vision, hallucinations were biased towards the larger objects (Figure 9C). Possibly, this is because larger shapes evoked higher overall activity in the model and in turn were more suitable for activity restoration (note for example in Figure 8A the transition from smaller to larger hallucinations as activity increases from point 3 to 4). On the contrary, in models with lesions restricted to the top half of the visual field, hallucinated objects were not only localised to the impaired region as reported above, but the frequency ratio was also reversed: smaller objects were much more common, and larger objects were less frequent and narrowly centred relative to the impaired region (Figure 9D). Moreover, we found that, without a single exception, *all* hallucinations of larger shapes happened to be of the ‘downwards-triangle’ category–the only large category where most of the object could fit into the lesioned region.

Thus, the process that generates hallucinations due to homeostatic adaptation can specifically evoke only certain types of content as determined by the nature of the visual impairment. Here, it is those objects that happen to fit within the boundaries of the lesion in the visual field.

### The locus of hallucinations: cortical lesions vs. suppression

We then turned our attention to the question of the roles of different areas in the cortical hierarchy. As described in the introduction, the complex content of hallucinations in CBS suggests the involvement of visual association cortex and other higher visual regions, and evidence implies that intact association cortex is both necessary and sufficient to develop complex hallucinations. For example, cortical lesions in early visual areas can bring about the visual impairment that causes complex hallucinations, but lesions that involve visual association cortex appear to prohibit them.

Interestingly however, a study by [69] suggests that lower areas, when at least partially intact, can still contribute to hallucinatory activity in an essential fashion. The authors examined a patient suffering from CBS due to visual impairment caused by lesions in early visual areas. Maybe contrary to expectation, applying Transcranial Magnetic Stimulation (TMS) to early areas in a way thought to cause cortical suppression lead to a temporary cessation of the hallucinations. The authors argue that their finding goes contrary to the ‘release’ theory of complex hallucinations, according to which the lack of input to higher areas from lower areas somehow disinhibits or releases perceptual representations. Under this theory, the further suppression of the already damaged early areas in the patient should only have exaggerated the hallucinations.

Using the DBM model, we examined these issues relating to the role of areas in the cortical hierarchy. The hierarchical computations in the DBM are simplistic compared to the cortical equivalent; however, we show that a generative model consisting of several subsequent processing stages differentiated at least by increasing receptive field sizes is sufficient to explain the phenomena at hand.

To begin with, we found that DBMs trained without the topmost hidden layer failed to learn generative models of the data, and thus were inevitably incapable of producing corresponding hallucinations. This mirrors visual association cortex being necessary for complex hallucinations, and can be explained in the model with lower layers being incapable of learning the full structure of objects in the images, due to their limited receptive field sizes.

What about intact higher areas being sufficient for the emergence of hallucinations, while lower ones are not necessary? To model lesions to early visual areas, we repeated the homeostasis experiment, only this time we did not blank the input but rather ‘lesioned’ the first hidden layer, i.e. we clamped units in the latter rather than the units in the visible layer to zero (thus, with the first processing stage blocked, the actual content in the visible units was rendered irrelevant). As before, hallucinations did emerge over the course of homeostatic adaptation (Supplementary Figure S3). Hence, remaining layers in the model are sufficient in principle as long as they form a network that can synthesise the relevant information about visual objects.

Finally, we modelled the suppression of early visual areas with TMS in a CBS patient as described by [69]. Unlike in the last experiment, where early areas were permanently incapacitated and higher areas adapted over time, the TMS experiment corresponded to a temporary suppression in a system that had already developed hallucinations, presumably due to prior adaptation to visual impairment. Our setup thus used a model that had undergone homeostatic adaptation in response to blank visual input but with all hidden layers intact, as in the first hallucination experiment, leading to hallucinatory activity. We then temporarily clamped the first hidden layer to zeros, modelling suppression with TMS (assuming that the cortical regions suppressed by TMS in the patient can be modelled to be downstream from the lesioned areas). This caused the hallucinations to cease. Thus, even though this ‘early area’ represented by the first hidden layer is neither sufficient nor necessary for the model to develop hallucinations in the long run (as shown earlier in this section), it can be essential for *ongoing* hallucinations if it was in the first place part of the system when it underwent homeostatic adaptation.

One possible interpretation of the relevance of lower areas could be that they provide higher areas with unspecific input, in the context of which the adaptation takes place. However, we suggest that the role of lower areas could be more subtle thanks to recurrent interactions with higher ones. As can be seen in the example in Figure 5, the representations assumed in lower layers during hallucinations are somewhat specific to the hallucinated object, even though those layers by themselves are incapable of synthesising it. Thus, this necessarily is a result of feedback from higher areas. It seems plausible that the lower areas could also contribute by stabilising the overall perceptual state assumed across the hierarchy. Then, any significant interference with representations in lower areas, not just suppression of activity, might impede hallucinations. Indeed, in the study of [69], even a TMS protocol used to cause not suppression but illusory flashes of light (“phosphenes”), applied to primary visual cortex of the patient, resulted in a disruption of hallucinatory content. In future work, this could be tested by trying out different forms of manipulations other than suppression in the hidden layers of the model.

### A novel model of acetylcholine and its role in CBS

Finally, one relatively common feature among CBS patients is that hallucinatory episodes are more likely to occur in states of drowsiness or low arousal. This suggests a role of cholinergic systems, which in turn are implicated in complex hallucinations in a variety of situations outside of CBS, whether drug induced or disease related [15], [30]. Indeed, in the (non-computational) model of complex hallucinations of [6], acetylcholine (ACh) dysfunction is attributed a major importance. At the same time, there is no evidence that an actual ACh dysfunction exists in CBS. Rather, in CBS the correlation with state of arousal might be effected by an interplay of hallucinations with physiologically normal fluctuations of ACh.

Making the connection between a lack of ACh and hallucinations is natural as there is experimental evidence that ACh acts specifically to emphasise sensory input over internally generated information, mediating “the switching of the cortical processing mode from an intracortical to an input-processing mode” [70]. In the computational model of [27], ACh is modelled in a Bayesian framework to modulate the interaction between bottom-up processing carrying sensory information and top-down processing conveying prior expectations. The authors noted the relation to hallucinations, but to our knowledge, there is no computational model exploring it concretely.

Here, we explore an extended interpretation of the action of ACh as mediating the balance between external and intracortical input: in the hierarchy of cortical areas, ACh could affect the balance in the integration of feedforward and feedback information at each stage of the hierarchy. At an intermediate stage, feedforward information from lower areas indirectly carries sensory input, and feedback information is more internally generated, keeping with the idea of a ACh mediated switch between external and internal inputs. However, both feedforward and feedback inputs would in this case be intracortical (perhaps with additional effects on any direct thalamic inputs).

We thus model the effect of ACh in the following way. In the DBM model, each (intermediate) hidden layer receives input from a layer below, conveying directly or indirectly sensory information, and from a layer above that has learnt to generate or predict the former layer's activity. ACh is to set the balance between feedforward and feedback flow of information. We introduce a balance factor 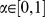, so that an intermediate layer 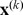 is sampled as
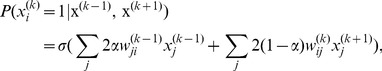
(6)given states 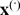 and weights 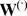 above and below (biases omitted for brevity). Hence, 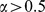 corresponds to increased feedforward flow of information, assumed to model increased ACh levels, and 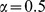 recovers the normal sampling mode for normal levels. We note that this mechanism is a heuristic in that it treats the DBM as a neural network more than a well-defined probabilistic model. In particular, for 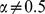, the effective connections between layers are no longer symmetrical and thus the model no longer constitutes a Boltzmann machine (in a sense, the factor 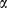 interpolates between inference in a DBM and approximate inference in a deep belief net [67], defined with the same parameters).

#### ACh and contour completion

Before we turn to the role of ACh in CBS, we first briefly demonstrate its effect on the balance between feedforward and feedback under normal sensory input. One example where it has been suggested that feedback could play a role is contour completion (see e.g. the hierarchical Bayesian inference account of [21]). Given an incomplete stimulus, higher areas might fill in missing information and subsequently convey it to lower areas, possibly leading to the perception of illusory contours.

**Figure 10:**
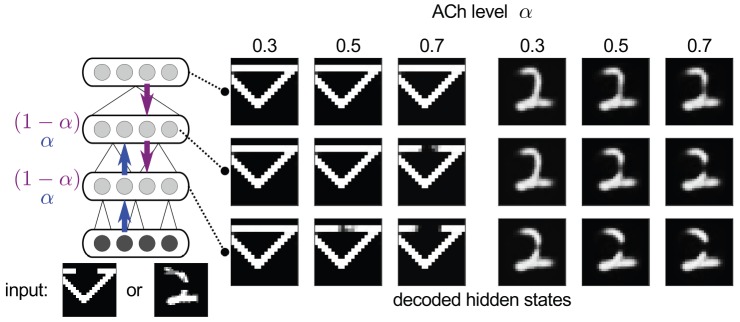
Contour completion and interaction with ACh levels 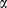. Incomplete images (lower left) were given to either shapes or MNIST models as input. Displayed are decoded hidden representations for the three hidden layers (rows), for three different levels of ACh (columns). Mean-field inference (i.e. propagating activities instead of samples [68]) was used here to reduce sample variability/noise. Filling-in of missing contours occurs more in higher than lower layers. ACh shifts the balance towards bottom-up processing, leading to less filling-in with increased levels.

We explored this phenomenon and a possible interaction with cortical ACh levels by testing the models on modified images where parts of the objects had been blanked out (Figure 10). Shown are examples of the decoded representations inferred by the model, for all three hidden layers and three different levels of ACh in both intermediate hidden layers, each for two different input images. We found that completion did indeed take place, especially in higher layers. Increased ACh levels, modelled with 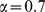, resulted in an emphasis on bottom-up processing, leading to less completion, in particular in lower layers that now received less top-down feedback. Decreased ACh levels on the other hand had the opposite effect. It should be noted that the generative nature of the DBM allows for much more extensive completion of image information if the visible units are sampled where filling-in should take place (e.g. [65]). In our model however, the whole visible layer always remains clamped, because this layer represents an early stage of processing where input is still represented faithfully in a bottom-up fashion. Filling in only happens in the subsequent hidden layers. Contour completion thus occurs more gradually in the hierarchy, rather than completely surmounting the sensory input itself.

#### ACh and CBS

We modelled the effect of drowsiness or low arousal on hallucinations in CBS as follows. We assumed that drowsiness is accompanied by a decrease in ACh, modelled as 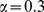. This value was chosen to obtain a clear effect while still allowing for both feedforward and feedback processing to play a role during inference. As states of drowsiness are intermittent with periods of normal or increased vigilance, we assumed that on average, ACh levels are still balanced. Hence, the homeostasis experiment was conducted such that at each iteration, activity levels were taken as average over 100 trials as before (see the Model section), but half of the trials were performed with low 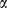, and the remainder with increased ACh levels at 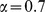, yielding a normal value of 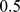 on average.

**Figure 11:**
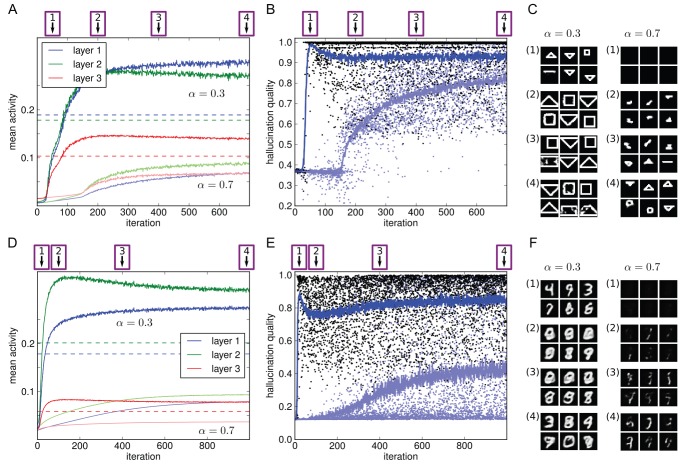
Hallucinations with fluctuating ACh levels. Over the course of homeostatic adaptation, each iteration consisted of both trials with low and high values of the ACh parameter, 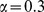 (dark curves, black dots) and 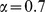 (light curves, light blue dots), respectively. (A–B) Average activities and hallucination quality for the shapes model. (C) Example decoded hallucinations at time points indicated in A–B. (D–F) Analogously for the MNIST model. For both models, lower ACh levels led to hallucinations earlier and with less homoestatic adaptation. In particular, there is an early phase in which hallucinations occurred only with 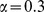. Hallucinations that do emerge later on for 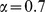 remain weaker and less formed for most of the simulation. The difference in frequency of hallucinations also entails a corresponding difference in activity levels.

For both shapes and MNIST models, results are displayed in Figure 11, both for trials with 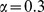 and 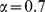 (dark and light curves, respectively). We found that with decreased levels of ACh, less homeostatic adaptation of excitability was necessary to elicit hallucinations (adaptation values not shown for brevity). In particular, for some intermediate level of adaptation, hallucinations only occurred with decreased but not with increased levels of ACh. This would thus correspond to a situation where hallucinations would only be triggered during drowsiness.

Throughout later parts of the simulation, activity levels for each hidden layer were generally twice as high during trials with 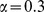 compared to those with 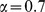, restoring original activity levels on average. Thus, alternating hallucinatory episodes and relatively silent periods, triggered by changing factors such as ACh levels, could restore mean activity levels, as long as the timescales over which neurons measure their average activity are long enough to encompass both. A possible prediction from our findings is that cortical activation during hallucinatory episodes should actually be higher than what they had been during healthy perception.

Hallucination quality actually peaked early on for low ACh trials, coinciding with the point in time when activity levels in those trials crossed approximately the original levels (point 1 in the figures). Because the neurons measured current activity over both low and high ACh trials, activity increased further, leading to a decreased quality of hallucinations. This was especially true for the MNIST model, where unnaturally high activity resulted in over-expressed imagery that showed little variety (point 2). However, over the further course of adaptation, activity levels for low ACh dropped again somewhat as the trials with higher ACh began to contribute activity, resulting in more distinct if still somewhat over-expressed hallucinatory images (point 4).

A related finding was a relationship between global activity levels and hallucinatory content in the shapes model. Corroborating what we observed earlier (see the experiment on localised hallucinations), internal representation of smaller shapes evoked less activation (averaged over a hidden layer) than that of larger shapes. Because alternating ACh levels led to hallucinations mostly during episodes of heightened activity, well-formed hallucinations developed to be mostly shapes of the larger categories. Increased activity levels thus caused hallucinations of larger extent in the shapes model and over-expressed digits in the MNIST model. Possibly, such over-activation of cortical neurons might explain why hallucinations in CBS can be so vivid, for example involving “hyperintense, vivid, brilliant colours” [71].

## Discussion

We modelled the emergence of complex hallucinations in CBS as a result of homeostatic regulation of neuronal firing rate in response to degradation of visual input. Our computational model thus elucidates on similar suggestions in the literature [12], [14]. The homeostasis mechanism is meant to underlie specifically CBS. Other pathologies involving complex hallucinations, such as schizophrenia or Lewy body dementia [15], might have different causes. In particular, it might not be feasible to unify complex hallucinations in a single explanatory framework (as proposed in [6]). What different conditions accompanied by complex hallucinations do have in common however is that they show that the brain can spontaneously synthesise rich representations of visual imagery, even in absence of or in contradiction to actual sensory data. Following notions of the brain implementing perception as analysis by synthesis, our study makes use of the DBM model that can learn to synthesise internal representations of images, in an unsupervised fashion, by virtue of being a generative model.

We reproduced a variety of qualitative aspects of CBS found in some patients, such as an initial latent period, a possible localisation of hallucinations to impaired parts of the visual field, and the effect of suppression of cortical activity. We predict a possible correlation between a tendency to experience miniature versions of objects and the degree to which the spatial extent of visual impairment is limited, as well as activity levels during hallucinatory episodes possibly being higher than what they had been during comparable, stimulus evoked normal perception. We introduced a novel model of the action of acetylcholine (ACh), suggesting that it could not only influence the balance between thalamic and intracortical inputs [70], but also the balance between feedforward and feedback at various stages of the cortical hierarchy. In CBS in particular, a possible lack of ACh at cortical sites, e.g. during normal fluctuations entailed in changes of state of arousal, could be conducive to the emergence of hallucinations.

We suggest that interfering with cortical homeostatic mechanisms might prevent the emergence of hallucinations in CBS. Whether such an intervention would be feasible in practice is unclear, given that the neurobiological mechanisms that underlie homeostatic plasticity are much more complex [33], [72] than our simple model of homeostatic adaptation. Alternatively, perhaps counter to intuition, it might be possible to suppress the formation of hallucinations in CBS by *up* regulating cortical activity in deprived areas, through pharmacological means or through methods such as TMS, as long as the externally imposed activation is too unspecific to allow for well-formed percepts to emerge.

In the model, internal representations of learnt objects were robustly recovered by the homeostatic adaptation in a variety of conditions, be it complete lack of input, noise input, or naturally structured but highly impoverished input consisting of fixed images. A key aspect of the model was that hallucinations did not consist only of stereotyped images, but rather a variety of percepts reflecting at least a part of the full distribution of objects learnt initially. Such variety across episodes is also reported in many CBS patients [2], [4]. In the model, this variability was due to different groups of neurons participating in coding for different percepts, meaning that a local homeostatic restoration of activity levels for the population required activation of a variety of percepts over time. We would predict that less variety in hallucinatory content should correlate with sensory deprivation being less extensive (e.g. only affecting colour vision, see below).

That hallucinations emerged even when normal input images were used but kept fixed over the course of homeostasis, shows that it was not so much the total lack of sensory input or global drop in evoked activity that mattered, but rather the failure of the given input to evoke a wide *range* of learnt percepts. Whether impoverished input can have such a powerful impact on perception in reality should be explored further. There is indeed evidence that sensory deprivation (in terms of general impoverishment, not just complete lack of sensory input) can cause hallucinations in healthy individuals [4], [29], but there seems to have been little experimental work along that direction since the nineteen sixties [73].

To our knowledge, our work constitutes the first computational model that concretely explored aspects of CBS. Other neurological pathologies have been studied before with neural network models [74]–[77]. Probably most closely related to our work, Ruppin et al. [53] modelled the emergence of hallucinatory memory patterns in schizophrenia, using a Hopfield network (a line of work initiated by [78]). The underlying mechanism, homeostatic plasticity in response to input degradation, is quite similar, and some analogous observations are made, including a beneficial role for homeostatic regulation for stabilising neuronal representations. However, in their model the hallucinatory ‘memories’, supposedly residing in prefrontal cortex, are accounted for much more abstractly, consisting of random patterns. Moreover, the retrieved patterns in a Hopfield net correspond directly to the patterns provided as input. It is thus not obvious how to relate their network and the stored patterns to specifically visual processing, which is essential for studying CBS. Our model can be seen as a significant extension of their work in that direction. It involves hierarchical, topographic representations of images, learnt in a generative model framework. In particular, the synthesised representations are interpreted to play an integral part in *perception* itself, not just in unspecified memory-like pattern recall. A generative model moreover relates to other approaches discussed in the context of hallucinations (Bayesian inference, predictive coding, adaptive resonance; [27]–[29], [79]).

We emphasise the distinction between the roles that homeostatic adaptation and learning play in our model and possibly the cortex. Learning is to be seen as a lasting change of circuitry that captures aspects of the sensory input in the neuronal representations, improving the network's function according to some criterion. In the generative model, that criterion would be the ability to generate or predict the input itself, but it could also be the utility of the representations towards some other goal, such as discrimination of objects. Homeostatic adaptation on the other hand could serve to stabilise neuronal representations. While such stabilisation can in turn be important during learning itself [33], we have shown in the model that it could offer a simple local mechanism to make representations more robust once they have been learnt, for instance to counteract degradation in input quality [53] –thus effectively resisting changing aspects of the input, rather than capturing them via learning.

At the point in time where we simulate homeostatic stabilisation, learning might have concluded, having taken place in earlier stages of development, or it could still occur but over longer time scales. A decoupling of the time scales of homeostatic adaptation and learning could also explain why CBS can recede over time. Hallucinations might initially be caused by the short-term homeostatic regulation of neuronal activity, but long-term cortical reorganisation could lead to their cessation [14]. In our framework, such reorganisation would correspond to learning to generate the *impaired* sensory input. Indeed, if we continue learning in the model as the input layer is clamped to empty or noise images, rather than just perform homeostatic adaptation, the model learns to generate and thus represent the empty input, losing the capability for hallucinations in the process.

### CBS in comparison to schizophrenia

In conditions such as schizophrenia, multiple sensory modalities are affected and hallucinations are only one symptom among many, including delusional beliefs. In contrast, hallucinations in CBS are, by definition, restricted to the visual modality and patients gain insight into the unreality of their percepts (at least upon reflection or after being corrected by others [2], [4]). These features of CBS are explained by our account: in our model, hallucinatory representations are restricted to neuronal populations most directly affected by lack of sensory drive (even respecting retinotopy). Thus, there is no reason to expect that non-visual areas should be impaired in any way, including prefrontal areas. CBS patients should hence be able to reason about their percepts being unreal.

As for the underlying mechanisms, we suggest that homeostatic compensation triggered by degrading input is key to CBS but not necessarily schizophrenia (though see [53]). Briefly, many neural network models of schizophrenia [76], [77] can be characterised as proposing that internal disruptive neural changes (such as increased noise or excessive synaptic pruning) *destabilise* internal representations, primarily in non-sensory areas or across cortical systems (thus affecting reasoning as well). In sensory areas deprived of sensory input, it is not clear that unspecific maladaptive changes such as increased noise alone could generate the lasting, complex, coherent, and varying hallucinations of CBS. Instead our proposal is that in CBS, it is in a sense a *stabilisation* of internal representations, in response to external disruptions in the sensory periphery, that causes hallucinations.

It should be noted that neurobiological changes such as increased noise or synaptic pruning could also be explored in the DBM. However, if non-sensory areas such as prefrontal cortex are the subject of inquiry, then the DBM and the hierarchical generative model it embodies might not be the most appropriate framework.

Our study can also be compared to recently proposed Bayesian accounts of schizophrenia [29], [77]. Hallucinations in CBS could on a high level be described as internal priors being too strong. Bayesian accounts of schizophrenia, however, involve more complex hypotheses about the role of feed-forward and feed-back processing (e.g. in the context of predictive coding [29]) that are not the focus of our study.

### Some open questions in CBS

One of the issues we have not addressed is what limits the incidence of complex hallucinations and CBS to about 11% to 15% of patients suffering from visual impairment [4]. Our modelling results suggest however that a variety of parameters can influence whether and when hallucinations occur. In the model, the nature and degree of visual impairment as well the effect and variability of other interacting factors, such as ACh levels, determine how much homeostatic adaptation is necessary to push cortical activity into the hallucinating regime. Limits on how much cortical neurons can adapt their excitability therefore would restrict hallucinations to only certain cases, and there might be variability in such parameters of homeostasis across the population as well. Thus, that only some patients with visual impairment develop hallucinations could simply reflect the variance of the underlying relevant parameters. Similar reasoning might explain the diversity of symptoms among CBS patients.

Differences in hallucinatory content, e.g. whether it does or does not involve movement, faces, strong colours, etc., likely relate to the specialisation of different cortical areas [3], [71], and potentially to their selective sensory deprivation (such as more extensive impairment of colour vision possibly predisposing patients with senile macular degeneration to experience coloured hallucinations [3]). A specialisation of different areas to different aspects of the sensory data was not a feature of our model. However, it seems reasonable to extrapolate from our results to a model extended in that regard. In our simulations, restricting sensory input by either removing only parts of the images or by just fixing input to a single image led to hallucinations that reflected the specific lack in the input (namely hallucinations in the deprived part of the visual field, or of object types not present in the fixed input image, respectively). If different parts of the model were to distinctly represent properties of visual input in analogy to for example cortical areas V4 for colour and MT for motion, we would expect a specific deprivation of that input property to lead to corresponding hallucinatory representations.

An open question in CBS is also in how far hallucinated content reflects visual memories of some sort [4], although the elaborate and occasionally bizarre nature of the images might speak against this (see [2], [12] for examples). In this context it is relevant that the DBM has been shown to be capable of synthesising images that generalise beyond what it has been trained on [65]. Moreover, in light of the bizarre or unusual hallucinatory imagery in CBS, some hallucinations with low quality in our simulations (as measured relative to training images) could possibly be interpreted as such unnatural imagery (see e.g. Figure 8b (3); Ruppin et al. [53] made a similar observation in their model).

### Challenges for a computational model of CBS

The key for a model of CBS is to account for the ability of the brain to synthesise rich internal representations of images even without visual input, representations that possibly generalise over earlier experienced inputs (as argued above). This does not *necessarily* imply that the brain implements a generative model, in the sense captured by the DBM. However, the strength of such generative frameworks is that they account for these aspects naturally, at least in principle.

For comparison, a perceptual Bayesian model defined over a single low-dimensional variable can be sufficient to account for perceptual *illusions* concerning a property of an object (e.g. due to a prior for slow speeds [80]), but it is far-off from actually generating a full visual representation of the object itself. Similarly, the necessity for synthesis without input implies that a model computing a rich *code* of a given image is on its own not sufficient either. For example, the predictive coding model of [18] and the sparse coding model of [81] are both formulated as generative models that learn representations from images. Given an input image, they can infer a code that is rich enough in information to reconstruct the former. However, neither model can, when run purely generatively, synthesise structured images or anything akin to objects (although [82] demonstrate that memorised images can be recalled). In particular, sparse coding trained on images tends to discover localised patches of edges as independent ‘causes’. Thus, without an extension to higher level causes, a generated image will be a random superposition of such edges.

Similarly, neural networks like (deep) auto-encoders learn internal representations by reconstructing input. Using bottlenecks in the hidden layers, sparsity, input reconstruction from noise-corrupted input and other techniques [37], they also learn about the underlying structure in images, enabling them to reconstruct from corrupted input, perform dimensionality reduction, or even learn transformations of the content [83]. However, there is no way of generating from these models in the absence of input (but see the recent work of [84]). Hence, again such an approach might be used to model illusions, but not hallucinations.

Clearly, while our model, the DBM, is a generative model, its capability to generate ‘images’ still leaves much to be desired when it comes to matching the perceptual richness attributed to real images (although the DBM and closely related models have shown more potential in that regard than what is demonstrated here, see [36], [85], [86]). As model of cortical representations and processing, it also makes several simplifying abstractions, such as lumping together the highly differentiated feedforward and feedback connections in the cortex (e.g. [87]) into simple symmetrical connections. Of particular interest are thus recent extensions that could enhance the generative performance of DBM-like approaches while at the same time having biological relevance as well, such as including lateral connections [88] or complex cell like pooling [89], [90].

However, our work here demonstrates that the DBM does in principle capture several aspects important for explaining CBS, idealisations notwithstanding. It is not meant as definitive model of generative processing in the brain, but rather serves as a simple idealised model system just complex enough to convey the points in question. Among the relevant aspects it captures is, first, the aforementioned capability to synthesise representations of input. Second, its hierarchical and topographic representations allowed us to model localised impairment and a role for ACh. Third, the nature of the DBM as a neural network made it possible to model concrete cellular homeostatic mechanisms. Fourth, unlike for example the earlier Helmholtz machine model [91], the DBM uses top-down interactions also during inference, not just learning, another requirement for modelling the role of hierarchical bottom-up and top-down processing for hallucinations. There are other aspects of cortical processing that are not part of the DBM framework but were not essential to the questions we sought to address in this work. The DBM would be less suitable if, for example, one were to hypothesise that some features of CBS relate specifically to the anatomical or functional asymmetry of cortical feedforward and feedback connections.

### ACh and probabilistic inference

Our model of the action of ACh is closely related in spirit to that of Yu and Dayan [27]. In a sense we addressed some of the issues they identified with their own approach, namely only dealing with a localist representation of a low-dimensional variable, and only with a shallow hierarchy where the interaction of bottom-up and top-down is confined to a single stage. As they write, “it would be more biologically realistic to consider distributed representations at each of many levels in a hierarchy”, which might be closer to what our model implements.

In Yu and Dayan's model, the ACh mechanism implements an approximation to exact inference: only a single hypothesis is maintained at any point in time by the top-down part of the system, with ACh controlling the impact of that hypothesis on perceptual inference. This is comparable to the action of ACh on the influence of higher layers on lower layers in our model. However, the functional role of ACh was not the main focus of our work, and in some ways their model is significantly more sophisticated than ours in that regard. In particular, in their model the ACh level is itself controlled by the system dynamically during ongoing inference, whereas we merely manipulated ACh manually to explore its impact on emerging hallucinations. Whether such an internal control of the ACh parameter 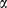 could be implemented in the DBM framework, in particular in a principled fashion, is open.

Another issue is in how far the role of ACh, and the interaction of top-down and bottom-up in hallucinations in general, is necessarily to be interpreted in ‘Bayesian’ or probabilistic terms. In Yu and Dayan's model, ACh represents the uncertainty associated with the current top-down hypothesis, and this uncertainty is itself subject to ongoing probabilistic inference. Because a mechanism for inferring this uncertainty is lacking in our model, we would be more cautious to necessarily frame the interaction of bottom-up and top-down as ‘Bayesian’ here. For our approach here, the probabilistic nature of the DBM only comes into play in so far as it allows for a means of formulating and deriving a generative model of sensory data (we emphasise the probabilistic aspect of the DBM model elsewhere [35], [52]).

### The nature of hallucinatory experience

A subtle issue is how much information needs to be synthesised in the brain, and in what form, to generate the visual experience of hallucinations. Mostly avoiding the difficult question of the neural correlates of consciousness here (e.g. [92]), we can at least pose necessary, though not sufficient, conditions for the generated neuronal representations to evoke complex visual hallucinations: they somehow must entail the information content that is implied in the percepts (assuming CBS patients are not just confabulating). For example, both seeing and hallucinating a dog entails much more than just being aware (and able to report) that the object in question is indeed a dog, i.e. some sort of category label. Rather, it involves perceiving the shape, contours, texture, colours, and so forth. Thus, internal activation of an abstract, low-dimensional representation of the concept of a dog would not be sufficient.

For instance, consider a simple perceptual model consisting of a neural network classifier such as a perceptron, which has learnt to classify images of dogs against other images, using a single binary output ‘neuron’. Internal activation of this unit alone cannot possibly be accompanied by the visual experience of seeing a dog, as the single bit of information conveyed by its state cannot possibly be used to differentiate among the various possible instantiations of dogs (a dalmatian in a specific pose rather than a poodle in another, etc.) [93].

The synthesis of rich internal representations of data, and how this capability is acquired through learning in the first place, is naturally explained in strong generative models such as the DBM. In the cortical hierarchy, a top-down generative component could also offer a mechanism to recover more detailed low-level representations from more high-level abstract representations, details that might be discarded during bottom-up or feedforward processing to obtain invariant representations (e.g. [94]). Alternatively, such detailed information might still be present at the high-level, but be only implicit and not easy to access by the rest of the brain. Top-down processing could then serve to transform such information into a more explicit (for the rest of the brain) representation. Either could explain why the generation of conscious experience might be related to re-entrant top-down processing [92].

### Conclusion

We have demonstrated how the DBM as a generative neural network can provide potential insights into the mechanisms underlying complex visual hallucinations in CBS. Our results here, together with other work [35], [52], [66], offer a novel perspective on perceptual phenomena by relating them to inference in a generative model in the cortex.

## Supporting Information

Figure S1 **Emergence of hallucinations due to homeostatic adaptation.** Equivalent to Figure 6 in the main text, but with a model trained on the shapes data set instead of MNIST. See main text for detailed explanation. (A–C) Removing the visual input led to a drop of activity levels and subsequent recovery through homeostatic adaptation (original activity levels as dashed lines). (D–F) Quality of hallucinations (i.e. how well decoded internal representations matched the learnt images), which emerged after an initial period of silence. 5 out of 100 trials per iteration are plotted. Blue curves denote mean quality over 100 trials in that iteration.
(EPS)

Figure S2 **Comparison of results of homeostatic adaptation for blank input images or noise images.** The noise images contained 10% white pixels on black background. Light curves and dark curves correspond to blank and noise images, respectively. (A–C) Results (activity changes and hallucination quality) for the model trained on shapes. (D–F) Results for the model trained on MNIST. For both models, noise input caused hallucinations to emerge after fewer iterations and with less adaptation of the biases. Moreover, comparing the differences in activity between first and second layer across conditions, it appears that the recovery of the first was less delayed relative to the second when the former was receiving noise input.
(EPS)

Figure S3 **Hallucinations for models that had their first hidden layer ‘lesioned’ (clamped to zero).** The first hidden layer was lesioned rather than the visible input layer, modelling damage to early cortical areas rather than prior in the visual pathway. Results are overall analogous to the latter case (see main text for explanation). (A–C) Results (activity changes and hallucination quality) for the model trained on shapes. (D–F) Results for the model trained on MNIST.
(EPS)

Text S1 **Learning.** A brief overview of how representations are learned in a DBM.
(PDF)

Text S2 **Additional training details.** Summary of training parameters used for learning the DBM model in this study.
(PDF)
