# Supplementary material for: Charles Bonnet Syndrome: Evidence for a Generative Model in the Cortex?
Source: PLoS Comput Biol. 2013 Jul 18;9(7):e1003134. doi: 10.1371/journal.pcbi.1003134 (PMC3715531; doi:10.1371/journal.pcbi.1003134)
Supplement: Text S1 — Learning. A brief overview of how representations are learned in a DBM. (PDF) [file pcbi.1003134.s004.pdf]

Charles Bonnet Syndrome: Evidence for a Generative Model in the Cortex?

Supplementary text S1: Learning

By interpreting a BM as defining a probabilistic model, learning can be formalised and derived as likelihood optimisation of the model parameters, given some observed data $\{\mathbf{v}^n\}_1^N$. The iterative weight update rule for general BMs is obtained by implementing (stochastic) gradient descent on the negative likelihood function. It turns out that the resulting algorithm involves a simple form of Hebbian (and anti-Hebbian) learning, changing the connections between any pair of units according to whether they are jointly active or not. Concretely, the incremental weight change is calculated as (the states of visible and hidden units are denoted by $\mathbf{v}$ and $\mathbf{h}$, respectively, and all states by $\mathbf{x}$, i.e. $\mathbf{x} = (\mathbf{v}, \mathbf{h})$):

$$\Delta w_{ij} \propto \sum_{n=1}^{N} \left\{ \langle x_i x_j \rangle_{P(\mathbf{h}|\mathbf{v}=\mathbf{v}^n)} - \langle x_i x_j \rangle_{P(\mathbf{h},\mathbf{v})} \right\} \tag{1}$$

Eq. 1 entails two expectation terms under the model distribution, given the parameters at that point in learning. The first is computed over the conditional distribution given the observed data, the second over the full joint distribution. Both terms are in general intractable for general BMs, thus approximation schemes are necessary, such as MCMC sampling as mentioned in the main text. In practice, the resulting algorithm then is as follows. In the 'positive phase', the first expectation is computed. The hidden units are activated by the input in the visible layer, sampling from the posterior during perceptual inference. In the 'negative phase', the second expectation is computed by sampling both hidden and visible units freely according to the internal model learnt by the machine so far. Both phases contribute the Hebbian weight changes that together realise learning.

The effect of the positive phase is that the model puts more probability mass on the observed sensory input. The effect of the negative phase is to reduce probability mass overall across the model distribution. In particular, this has the net effect of removing probability mass from regions in the input space which are not supported by the observed data. The offline generation of model 'fantasies' in the negative phase data suggests the metaphor of dreams. Perhaps the metaphor can be taken more seriously, leading to the hypothesis that the role of dreaming could be the removal of spurious modes learned by the brain's internal model [1].

The basic BM learning algorithm usually requires infeasibly long sampling to produce useful gradient estimates. Three key points allowed for BM-based models to be increasingly used in machine learning over recent years. First, research has focused on BMs with simplified connectivity, primarily the Restricted BM (RBM), which has no connections between visible nor between hidden units (a 2-layer DBM is a RBM). In the RBM, hidden units are conditionally independent given the visible units (meaning they do not interact if the visible units are fixed e.g. to input data). This renders inference and with it the positive phase of the weight update straightforward, though the negative phase can still be problematic. The second key was thus the development of more effective approximations to the standard learning algorithm, most prominently the Contrastive Divergence (CD) algorithm [2]. In a rather crude [3] approximation to the original negative phase, CD replaces a set of samples representative of the full model distribution with a single sample close to the data point currently utilised in the positive phase. Recently it has been argued that CD could be understood as a form of reconstruction error driven learning [4], which would at least in spirit relate it to models of cortical inference and learning such as predictive coding [5–7]. Another relevant approximate training algorithm is Persistent Contrastive Divergence (PCD) [8,9], which like CD only uses few samples and sampling steps in the negative phase, but unlike CD decouples the negative phase from the positive phase. Elsewhere we have shown how an extension of PCD can be interpreted as a biological sampling algorithm that utilises neuronal adaptation [10].

A third key development was then to return to more powerful architectures such as the DBM, still making use of the simpler RBM in the process [11, 12]. Similarly to several other Deep Learning models [13], the DBM is initially trained *one layer at a time* such that each layer learns to generative the activity patterns in the layer below [14]. To this end, each adjacent pair of layers is treated as RBM. This iterative learning along the hierarchy might be reminiscent of the sequential maturation of areas in the cortical hierarchy, especially for the ventral visual stream [15]. Once the full DBM is composed, further learning can take place, e.g. using additional techniques such a mean-field inference.

Importantly, in all theses cases, whether it is the original BM learning algorithm, approximations such as CD, or the training of DBMs, the involved computations again turn out to have 'neural' interpretations (see [16] for an extended discussion). Hence, the general learning principles the DBM and similar models are based on–such as unsupervised learning employing a generative model, iterative learning of a hierarchy, perhaps unlearning of spurious modes–could make for interesting hypotheses about the cortex. At the same time, the concrete implementation details, while necessarily deviating from any more biologically realistic implementation in the cortex, are all based on simple mechanisms such as Hebbian learning, at least posing no implausible challenges for biological analogues or substitute mechanisms in the brain.

Finally, it should be noted that DBMs and similar models can learn receptive fields reminiscent of those of V1 neurons, and possibly of V2 neurons [17]. This is a property common to several learning algorithms [18], especially if they enforce some notion of sparsity on the activation levels, as with the original sparse coding algorithm of Olshausen and Field [19].

References

1. Crick F, Mitchison G (1983) The function of dream sleep. Nature 304: 111–114.

2. Hinton GE (2002) Training products of experts by minimizing contrastive divergence. Neural Computation 14: 1771–1800.

3. Hinton GE (2010) A practical guide to training restricted Boltzmann machines. Technical report UTML TR 2010-003, Department of Computer Science, Machine Learning Group, University of Toronto.

4. Bengio Y, Delalleau O (2009) Justifying and generalizing contrastive divergence. Neural Computation 21: 1601–1621.

5. Mumford D (1992) On the computational architecture of the neocortex. Biological Cybernetics 66: 241–251.

6. Rao RP, Ballard DH (1999) Predictive coding in the visual cortex: a functional interpretation of some extra-classical receptive-field effects. Nature Neuroscience 2: 79–87.

7. Friston K, Kiebel S (2009) Predictive coding under the free-energy principle. Philosophical Transactions of the Royal Society B: Biological Sciences 364: 1211 –1221.

8. Tieleman T (2008) Training restricted Boltzmann machines using approximations to the likelihood gradient. In: Proceedings of the 25th Annual International Conference on Machine Learning. Helsinki, Finland, pp. 1064–1071. doi:10.1145/1390156.1390290. URL `http://portal.acm.org/citation.cfm?id=1390156.1390290`.

9. Younes L (1989) Parametric inference for imperfectly observed Gibbsian fields. Probability Theory and Related Fields 82: 625–645.

10. Reichert DP, Seriès P, Storkey AJ (2011) Neuronal adaptation for sampling-based probabilistic inference in perceptual bistability. In: Shawe-Taylor J, Zemel R, Bartlett P, Pereira F, Weinberger K, editors, Advances in Neural Information Processing Systems 24. pp. 2357–2365.

11. Hinton GE, Osindero S, Teh YW (2006) A fast learning algorithm for deep belief nets. Neural Computation 18: 1527–1554.

12. Hinton GE, Salakhutdinov RR (2006) Reducing the dimensionality of data with neural networks. Science 313: 504–507.

13. Bengio Y (2009) Learning deep architectures for AI. Foundations and Trends in Machine Learning 2: 1127.

14. Salakhutdinov R, Hinton G (2009) Deep Boltzmann machines. In: Proceedings of the 12th International Conference on Artificial Intelligence and Statistics (AISTATS). volume 5, pp. 448–455.

15. Bourne JA, Rosa MG (2006) Hierarchical development of the primate visual cortex, as revealed by neurofilament immunoreactivity: Early maturation of the middle temporal area (MT). Cerebral Cortex 16: 405 –414.

16. Reichert DP (2012) Deep Boltzmann Machines as Hierarchical Generative Models of Perceptual Inference in the Cortex. PhD thesis, University of Edinburgh, Edinburgh, UK.

17. Lee H, Ekanadham C, Ng AY (2008) Sparse deep belief net model for visual area V2. Advances in Neural Information Processing Systems 20 .

18. Saxe AM, Bhand M, Mudur R, Suresh B, Ng AY (2011) Unsupervised learning models of primary cortical receptive fields and receptive field plasticity. In: Shawe-Taylor J, Zemel RS, Bartlett P, Pereira FCN, Weinberger KQ, editors, Advances in Neural Information Processing Systems 24. pp. 1971–1979.

19. Olshausen BA, Field DJ (1996) Emergence of simple-cell receptive field properties by learning a sparse code for natural images. Nature 381: 607–609.
