# Supplementary material for: Charles Bonnet Syndrome: Evidence for a Generative Model in the Cortex?
Source: PLoS Comput Biol. 2013 Jul 18;9(7):e1003134. doi: 10.1371/journal.pcbi.1003134 (PMC3715531; doi:10.1371/journal.pcbi.1003134)
Supplement: Text S2 — Additional training details. Summary of training parameters used for learning the DBM model in this study. (PDF) [file pcbi.1003134.s005.pdf]

Charles Bonnet Syndrome: Evidence for a Generative Model in the Cortex?

Supplementary text S2: Additional training details

For training, we used either CD-1 or PCD-5 (see Supplementary Text S1) for layer-wise pre-training, where each pair of adjacent layers forms a RBM, and no fine-tuning of the full DBM. It should be noted that learning in BM type models can be somewhat of a (black?) art, as there are various meta-parameters that affect the outcome, such as the number and sizes of the hidden layers and the learning rate, and only heuristic recipes for setting them [1]. There are also additional tricks like using momentum (meaning that the weight updates are smoothed by averaging the current gradient with recent ones) and weight decay [1]. For our work, we based our initial implementation on publicly available deep belief net code [2], and kept meta-parameters in default ranges unless a change was necessary (e.g. when implementing PCD over CD). In general, our study was concerned with simulating qualitative phenomena rather than with breaking performance benchmarks or matching quantitative experimental data. To achieve the results to be presented, we found that little parameter fiddling was necessary once a model setup worked in principle.

Below we list the parameters values used for training the RBM components of the DBM. Details of the model architectures and data sets are described in the main text. The general procedures and parameter ranges adhered to the 'practical guide' to training RBMs of [1], to which the reader should refer to for further elaboration. The underlying concepts are also explained in [3], with an emphasis on the biological relevance. CD-1 or PCD-5 were used depending on what was found to work better for the data set in question. Depending on the training variant, some training parameters differed: it was important to adjust the order of magnitude of the learning rate and the momentum meta-parameter to make the two methods work. Other differences had only a smaller impact, and were in part incidental aspects of exploring different training setups at the time.

All training data sets had 60,000 images and were split into minibatches of 100 images each. At the beginning of training, the weights were initialised randomly using values drawn from a zero-mean Gaussian with a standard deviation of 0.1. All biases were initialised to -4 to encourage sparsity. Training then proceeded over 30 epochs (i.e. iterations through the training data). Weights were updated using weight-decay with a weight-cost of 0.0002. For CD-1, the learning rate was kept fixed at 0.1; initial momentum was 0.5 for the first six epochs and then changed to 0.9. In the case of PCD on the other hand, the learning rate was initialised to 0.005 and then decreased linearly to 0 over the course of training; momentum for PCD was kept at 0.5 throughout; the Markov chain was run for 5 steps in the negative phase, with a number of fantasy particles matching the size of a minibatch (i.e., 100); and, activation probabilities were used for the visible states in the negative phase rather than sampled binary states. Lastly, to train the next RBM in the DBM, activation probabilities of the current topmost hidden layer were used as data.

References

1. Hinton GE (2010) A practical guide to training restricted Boltzmann machines. Technical report UTML TR 2010-003, Department of Computer Science, Machine Learning Group, University of Toronto.

2. Hinton GE, Salakhutdinov RR (2006) Reducing the dimensionality of data with neural networks. Science 313: 504–507.

3. Reichert DP (2012) Deep Boltzmann Machines as Hierarchical Generative Models of Perceptual Inference in the Cortex. PhD thesis, University of Edinburgh, Edinburgh, UK.
